# Trunk Muscle Activity and Ratio of Local Muscle to Global Muscle Activity during Supine Bridge Exercises under Unstable Conditions in Young Participants with and without Chronic Low Back Pain

**DOI:** 10.3390/healthcare12050514

**Published:** 2024-02-21

**Authors:** Akimi Nakata, Satoshi Osuka, Tomoya Ishida, Yuki Saito, Mina Samukawa, Satoshi Kasahara, Yuta Koshino, Naoki Oikawa, Harukazu Tohyama

**Affiliations:** 1 Faculty of Health Sciences, Hokkaido University, Sapporo 060-0812, Japan; 2 Department of Rehabilitation, Hokkaido University Hospital, Sapporo 060-8648, Japan; 3 INTAGE Healthcare Inc., Chiyoda-ku, Tokyo 101-0062, Japan; 4 Department of Rehabilitation, Faculty of Health Sciences, Japan Health Care University, Sapporo 062-0053, Japan

**Keywords:** low back pain, electromyography, exercise therapy, rehabilitation, unstable condition, bridge exercise

## Abstract

Core exercises on an unstable surface increase trunk muscle activity, especially for local muscle groups. Therefore, there is a possibility that exercises on an unstable surface would be effective in the rehabilitation of non-specific chronic low back pain (NSCLBP). The present study assessed trunk muscle activities during bridge exercise on the floor and two kinds of unstable surfaces, i.e., a balance ball and the BOSU, for individuals with and without NSCLBP. This study enrolled 17 and 18 young participants with and without NSCLBP, respectively. In the balance ball condition, both groups showed a significant increase in erector spinae activity compared to the floor condition, and the increase in activity was significantly greater in the NSCLBP group than in the control group (*p* = 0.038). On the other hand, neither group showed significant changes in trunk muscle activities in the BOSU condition compared to those in the floor condition. The control group showed a significant increase in internal oblique/transversus abdominis activity under the balance ball condition (*p* = 0.020), whereas there were no significant changes in these muscle activities between the balance ball and floor conditions in the NSCLBP group. The present study showed that participants with NSCLBP significantly increased muscle activity of the erector spinae, one of the global back muscles, on the balance ball in spite of small effects on muscle activity of the internal oblique/transversus abdominis, which is one of the local abdominal muscles. Therefore, attention should be paid to the application of bridge exercises on the balance ball for individuals with NSCLBP.

## 1. Introduction

Low back pain is one of the most prevalent and expensive musculoskeletal disorders worldwide [1,2]. Particularly, non-specific low back pain is defined as low back pain (LBP) that cannot be associated with an identifiable etiologic pathology, such as nerve root compromise or significant spinal pathology (i.e., fracture, cancer, and inflammatory diseases), and has been identified as the most prevalent type of LBP [3,4]. Patients with non-specific acute LBP have a favorable rate of improvement within the first six weeks [5]; however, approximately 40% of patients develop non-specific chronic LBP (NSCLBP) [6].

NSCLBP causes dysfunction of trunk muscles, and dysfunction of trunk muscles can also cause chronicity [7,8,9,10]. Trunk muscles are divided into local (e.g., transversus abdominis and multifidus) and global (e.g., erector spinae, rectus abdominis, internal and external obliques, quadratus lumborum, gluteus maximus, and latissimus dorsi) muscles based on functional differences [11]. Previous studies showed that individuals with LBP have impaired control of trunk muscles essential for maintaining the coordination and stability of the spine. A delayed onset of the abdominal local muscles associated with upper and lower limb raising movements was reported in patients with NSCLBP compared to the healthy control group [12,13,14]. In addition, Miura et al. evaluated the activity of the transversus abdominis muscle using an ultrasound imaging system and showed that the change in activity associated with postural change was significantly smaller in the NSCLBP group than in healthy individuals [7]. In contrast, global muscles in patients with LBP exhibit excessive muscle activity [10,11,12], which increases trunk stiffness [15,16]. Such motor control strategy activating global muscles employed in NSCLBP may be protective, aimed at reducing the induction of pain and injury to the spine and surrounding tissues. However, in the long term, these inappropriate muscle activity patterns are thought to contribute to NSCLBP [9]. In the rehabilitation of NSCLBP, it is recommended to increase the activity of local muscle groups [17]. Previous studies have stated that stabilization exercises for patients with NSCLBP should work local and global muscles in a coordinated manner [18,19].

Most clinical guidelines recommend exercise therapy as a treatment for NSCLBP since it reduces pain and improves function [20,21,22]. Particularly, exercises in unstable conditions are frequently performed for trunk muscle training in sports and rehabilitation [23,24]. Unstable foot support using a balance ball has been shown to increase the activities of the lumbar iliopsoas, lumbar multifidus, rectus abdominis, and external oblique abdominal muscles during supine bridge exercises in healthy individuals [25]. Imai et al. also found that abdominal and back muscle activities were significantly higher during the prone bridge, side bridge, hand–knee exercise, and curl-up exercises on the BOSU balance trainer than on the floor surface in healthy young adults [26]. Additionally, Czaprowski et al. reported that supine and prone bridge exercises on a balance ball resulted in higher abdominal muscle activity than that on the BOSU balance trainer and stable floor surface in healthy individuals [27]. Therefore, trunk muscle exercises on unstable surfaces may be effective in the rehabilitation of NSCLBP. However, no studies have examined trunk muscle activity during exercise in an unstable condition in patients with NSCLBP. Because individuals with NSCLBP exhibit different local and global muscle activities compared to those without NSCLBP, the changes in trunk muscle activity during exercises in unstable conditions may differ between individuals with and without NSCLBP.

The aim of this study was to compare trunk muscle activities in patients with NSCLBP with those in healthy controls during supine bridge exercises under unstable conditions using a balance ball and the BOSU balance trainer, which are widely used in exercise therapy for LBP [25,26,27]. In addition, the ratio of local muscle activity to global muscle activity in individuals with NSCLBP was also compared with that in healthy controls to investigate whether global and local muscles work in a coordinated manner. We hypothesized that the individuals with NSCLBP would show more activity in the global muscles and less change in local muscle activity than the control group during supine bridge exercises under unstable conditions and the ratio of local muscle activity to global muscle activity would be smaller in participants with NSCLBP than in healthy participants under unstable conditions.

## 2. Materials and Methods

### 2.1. Participants

**Figure 1:**
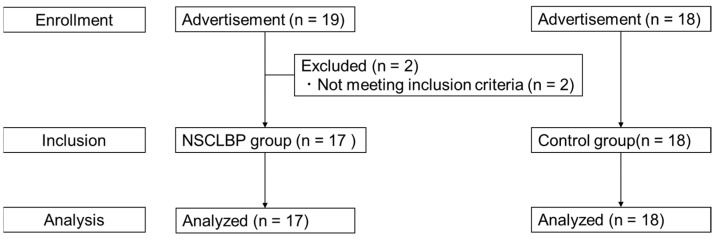
Flow chart for this study. We recruited 19 participants in the NSCLBP group and 18 in the control group; 2 were excluded because they did not match the inclusion criteria, 17 were assigned to the NSCLBP group, and 18 to the control group for analysis of trunk muscle activity.

Seventeen individuals with NSCLBP and eighteen healthy individuals participated in this study (Figure 1). The mean values of age, body height, body weight, and body mass index (BMI) for the NSCLBP group in this study were 21.8 ± 2.6 years, 166.5 ± 6.5 cm, 60.6 ± 7.8 kg, and 21.8 ± 1.9 kg/m^2^, respectively. Conversely, the mean values of age, body height, body weight, and BMI in the control group were 21.6 ± 1.0 years, 164.8 ± 8.3 cm, 56.3 ± 8.2 kg, and 20.6 ± 1.9 kg/m^2^, respectively. There were 10 male participants in the NSCLBP group and 8 male participants in the control group. A priori power analysis was performed in G*power 3.1 (University of Dusseldorf, Dusseldorf, Germany) using the activity of erector spinae in a previous study [28], and we calculated Cohen’s *d* = 1.01 as the effect size. In order to obtain a statistical power of 80% with an α level of 0.05 for the unpaired *t*-test, at least 17 participants were required. All participants were recruited by each advertisement for NSCLBP and control group at two universities. Participants with NSCLBP were recruited if they experienced pain between the 12th thoracic vertebra and the level of the coccyx for more than three months [10]. Healthy individuals were established as the control group, comprising individuals who did not exhibit symptoms of low back pain. The exclusion criteria for both NSCLBP and healthy control groups were the presence of severe spine pathology, neurological disorders, a history of fractures or surgery in the trunk or hip joints, pregnancy in the previous two years, and a history of physical therapy for the trunk [10]. The study was performed in accordance with the Declaration of Helsinki. The study was approved by the Institutional Review Boards of the Faculty of Health Sciences at Hokkaido University (approval number 21-33), and all participants provided written informed consent.

### 2.2. Experimental Procedures

This study was a case-control study in which trunk muscle activity was measured during supine bridge exercises in three different conditions, and the measurements were compared between groups. Participants in the NSCLBP group completed a questionnaire prior to the experimental session to assess the pain intensity and level of disability. We incorporated the numeric rating scale (NRS) and the Oswestry disability index 2.0 (ODI) into the questionnaire. The NRS was evaluated to investigate pain intensity by obtaining score reports from 0 (no pain) to 10 (excruciating pain). The ODI was used to investigate the effects of LBP on the activities of daily living. The ODI was developed to assess the degree of disability of patients with chronic LBP in activities of daily living and is the most widely used patient-reported outcome measure [29]. The ODI consists of 10 questions, each rated on a 6-point scale (0–5). The score is out of 50 points, with higher scores indicating greater disability. Participants with NSCLBP rated their symptoms for the past week.

**Figure 2:**
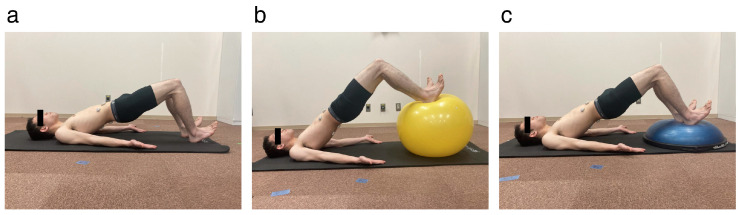
Bridge exercise (**a**) on the floor, (**b**) on the balance ball, and (**c**) on the BOSU balance trainer. The participants were barefoot, and their feet were supported only by their heels. They were instructed to keep their hips in a neutral position and avoid using arm strength during the exercise.

Bridge exercises in the supine position were used to assess the effect of unstable surfaces on trunk muscle activities [25,27]. Three surface conditions for the foot support were performed: the floor, a balance ball with a diameter of 65 cm (JOINUS La-VIE, Tokyo, Japan), and a BOSU (BOSU^®^ Pro Balance Trainer, BOSU, Ashland, OH, USA) (Figure 2). The balance ball and BOSU were placed under the heels. The participants were placed in a hook-lying position, which was in the supine position with the knees bent 90°, the feet supported on the heels only, and the hands on the floor with palms facing upward for all conditions [27]. Knee joint flexion angle during bridge exercises was confirmed by an examiner using a goniometer. The feet were barefoot and supported only by the heels [27]. The pelvis was then elevated until the hip joint flexion/extension was in a neutral position without the application of arm strength. Before the measurement, the participants were given a comprehensive explanation of the task. Feedback was given throughout the task to maintain correct posture without swaying in the neutral position of hip joint flexion/extension. Three trials were conducted for each condition. The three types of bridge exercises were performed in a randomized order.

### 2.3. Electromyography Evaluation

Electromyography (EMG) recordings of five trunk muscles: the internal oblique/transversus abdominis, external oblique, rectus abdominis, erector spinae, and lumbar multifidus muscles were recorded using a wireless surface EMG system (WEB-1000; Nihon Kohden Corporation, Tokyo, Japan) with surface-type electrode telemeters sampled at a rate of 1000 Hz. All muscle activities were measured on the right side. The attachment positions of each electrode were according to previous studies [26,30,31]: internal oblique/transversus abdominis, 2 cm medial and caudal to the anterior superior iliac spine; external oblique, midway between the costal margin of the ribs and the iliac crest, approximately 45° to the horizontal; rectus abdominis, 3 cm lateral to the umbilicus; erector spinae, 3 cm lateral to L3 spinous processes; lumbar multifidus, 2 cm lateral to L4–L5 spinous processes. Surface electrodes were placed parallel to the muscle fibers. Before applying the electrodes, the attachment position was skin-treated with alcohol and an abrasive.

### 2.4. Maximum Voluntary Isometric Contraction Trials

Following data acquisition of bridge exercises, maximum voluntary isometric contraction (MVIC) trials were recorded. In measuring the MVIC of each muscle, the participants were well-rested to minimize the effects of fatigue. MVIC data were obtained in a manner consistent with that reported in a previous study [26]: erector spinae and lumbar multifidus, prone position with the trunk fully extended and hands clasped behind the head, with resistance at the thorax in the direction of trunk flexion; internal oblique/transversus abdominis, supine position with hips and knees flexed, feet supported, and trunk maximally flexed and rotated to the right, with resistance at the shoulders in the trunk extension and left rotation directions; external oblique, supine position with hips and knees flexed, feet supported, and trunk maximally flexed and rotated to the left, with resistance at the shoulders in the trunk extension and right rotation direction; rectus abdominis, body supine with hips and knees flexed, feet supported, and trunk maximally flexed, with resistance at the shoulders in the trunk extension direction. During the assessment of MVICs, the duration of muscle contraction was intentionally limited to a brief period of 3 s. This was conducted in order to reduce the negative effects of longer contractions, such as demotivation and pain [32]. These MVIC measurements were performed in two trials.

### 2.5. Data Analysis

MATLAB 2019a (MathWorks, Natick, MA, USA) was used for data processing. All raw EMG signals were filtered using a bandpass filter with cut-off frequencies of 20–500 Hz. We counted five seconds while the participants maintained the correct posture and calculated the root mean square (RMS) using three seconds as the analysis interval. The data obtained in the balance ball and BOSU conditions were normalized by dividing them by the value of the data obtained on the floor. The mean value of three trials was calculated for each condition. Additionally, to determine whether trunk muscle activity is increased by the unstable condition, we also calculated values normalized by MVIC for each muscle activity on the floor, the BOSU, and the balance ball. The RMS for MVIC was calculated with one second as the analysis interval. The mean value of two trials was calculated for the MVIC. MVIC-normalized values were used to calculate the ratio of local muscle activity to global muscle activity by dividing local muscle activity by global muscle activity [19]. We calculated the ratio of internal oblique/transversus abdominis muscle activity to rectus abdominis muscle activity, the ratio of internal oblique/transversus abdominis muscle activity to external oblique muscle activity, and the ratio of lumbar multifidus muscle activity to erector spinae muscle activity [19].

### 2.6. Statistical Analyses

IBM SPSS Statistics 28 (IBM, Chicago, IL, USA) was used for statistical analyses. The Shapiro–Wilk test was used to examine normality. According to the normality, an independent *t*-test or Mann–Whitney U test was used for between-group comparisons of demographic data and trunk muscle activity normalized by floor supine bridge exercise under balance ball and BOSU conditions and the ratio of local muscle activity relative to global muscle activity. Additionally, to determine whether the unstable condition increased trunk muscle activity, each muscle activity in the MVIC-normalized balance ball and BOSU conditions was compared to the floor condition using either a paired *t*-test or a Wilcoxon signed-rank test. Additionally, the effect sizes for comparison of demographic data and muscle activity were calculated as Cohen’s *d* when the data followed a normal distribution and as r when the data did not adhere to a normal distribution. This was performed to quantify the extent of the differences. The effect sizes were categorized as small (*d* = 0.2 or *r* = 0.1), moderate (*d* = 0.5 or *r* = 0.3), or large (*d* = 0.8 or *r* = 0.5) [33].

## 3. Results

There was no significant difference in age, height, weight, or BMI between the NSCLBP and control groups (Table 1). The mean (standard deviation) scores for NRS and ODI in the NSCLBP group were 3.1 (1.7) and 14.2 (9.2), respectively (Table 1).

**Figure 3:**
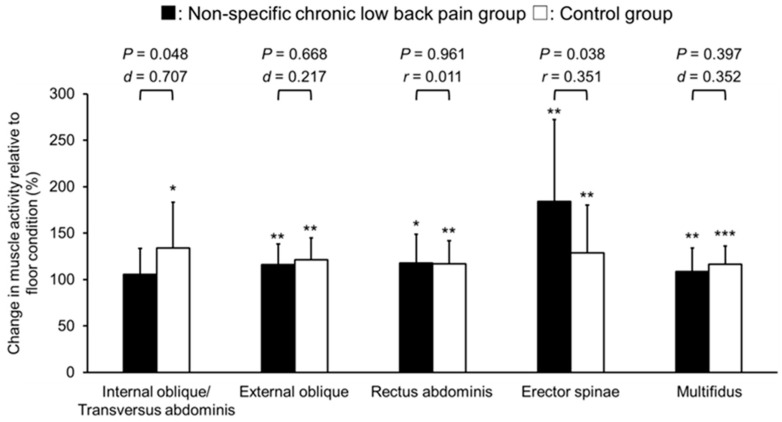
The trunk muscle activity normalized by floor bridge exercise under the balance ball condition in non-specific chronic low back pain and control groups. Significant differences between the balance ball and floor conditions are indicated by asterisks (*** *p* < 0.001; ** *p* < 0.01; * *p* < 0.05).

The control group showed significantly larger activities of internal oblique/transversus abdominis muscles in the balance ball condition than in the floor condition with a large effect size (*p* = 0.020, *r* = 0.549), whereas the NSCLBP group did not show significant change in internal oblique/transversus abdominis muscle activities between the floor and balance ball conditions (*p* = 0.055, *r* = 0.465). A significant group difference was found in the relative changes in the internal oblique/transversus abdominis muscles activities from the floor to the balance ball condition with moderated to large effect size (*p* = 0.048, *d* = 0.707) (Figure 3). Both groups showed significantly larger activities of erector spinae muscle in the balance ball condition than in the floor condition with large effect sizes (NSCLBP: *p* = 0.003, *r* = 0.729; control: *p* = 0.001, *d* = 0.760). Moreover, the increase in the erector spinae muscle activities from the floor condition to the balance ball condition was significantly larger in the NSCLBP group than in the control group, with a moderate effect size (*p* = 0.038, *r* = 0.351) (Figure 3). Both groups also showed significant increases in the activities of the external oblique abdominis, rectus abdominis, and multifidus muscles with moderate to large effect sizes between the floor and balance ball conditions (Figure 3, Table 2), while there were no significant group differences in the changes of those muscle activities from the floor condition to the balance ball condition (Figure 3).

**Figure 4:**
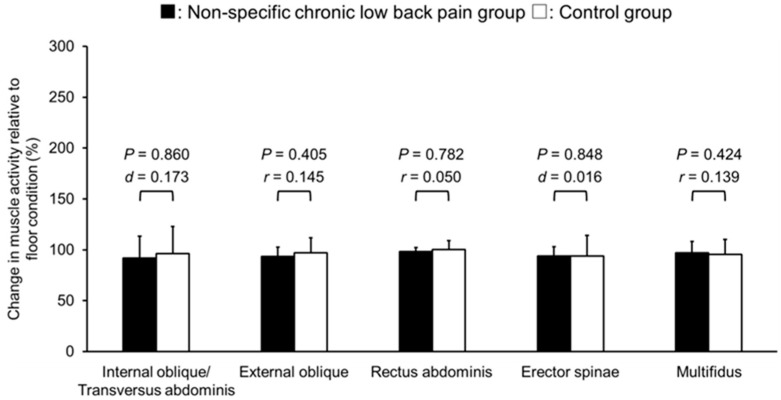
The trunk muscle activity normalized by floor bridge exercise under the BOSU condition in non-specific chronic low back pain and control groups. No significant difference was found between the BOSU and floor conditions.

In the BOSU condition, both groups did not show significant changes in trunk muscle activities compared to the floor condition, and all effect sizes were small (NSCLBP: *p* = 0.270–0.922, control: *p* = 0.349–0.682) (Figure 4). The relative changes between the floor and the BOSU conditions were not significantly different between the NSCLBP and control groups (*p* = 0.405–0.860).

**Figure 5:**
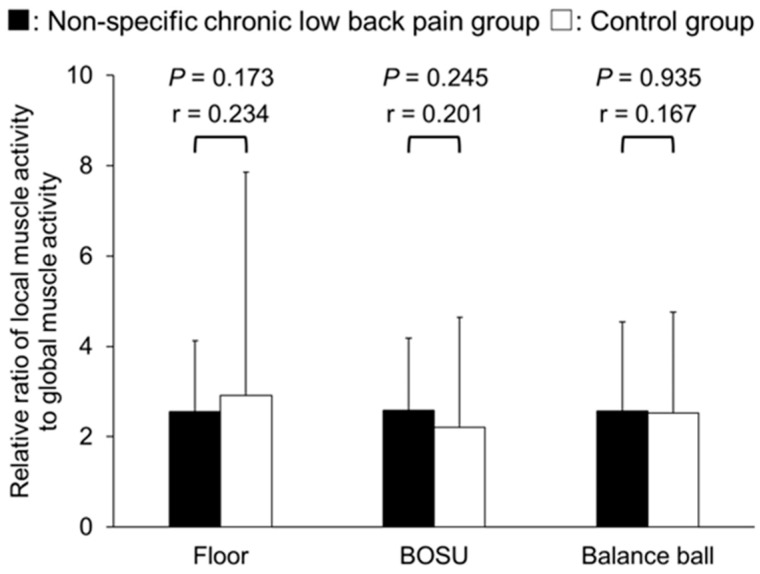
Relative ratio of external oblique muscle activity divided by internal oblique/transversus abdominal muscle activity.

**Figure 6:**
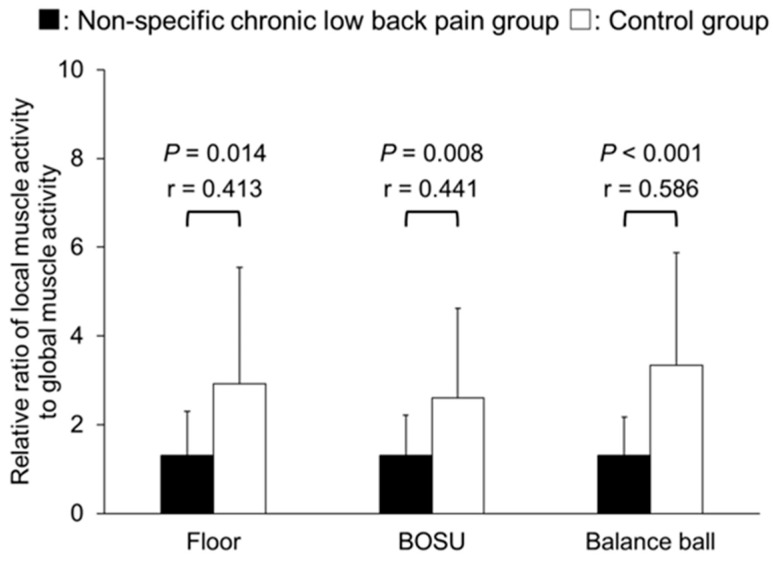
Relative ratio of rectus abdominis muscle activity divided by internal oblique/transversus abdominis muscle activity.

**Figure 7:**
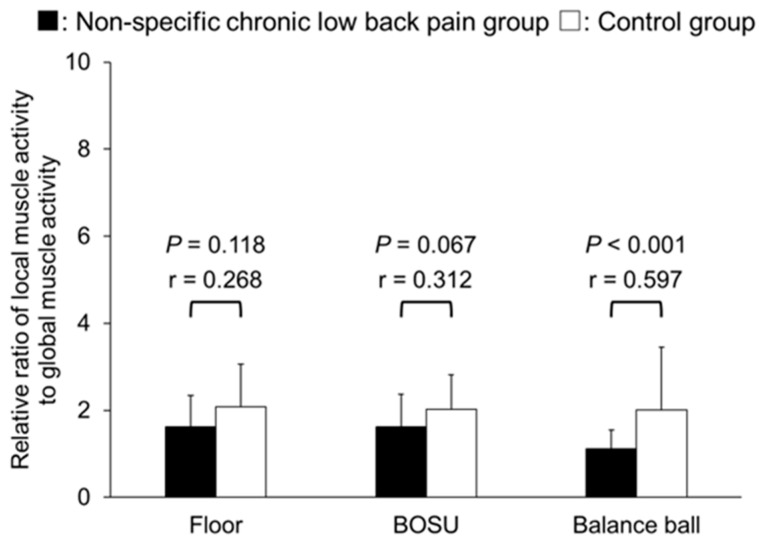
Relative ratio of erector spinae muscle activity divided by lumbar multifidus muscle activity.

In the ratio of local muscle activity to global muscle activity, no significant differences were found between NSCLBP and the control group in the ratio of internal oblique/transversus abdominis muscle activity to external oblique muscle activity in all conditions (*p* = 0.173–0.935), and all effect sizes were small (*r* = 0.167–0.234) (Figure 5). On the other hand, the ratio of internal oblique/transversus abdominis muscle activity to rectus abdominis muscle activity was significantly lower in the NSCLBP group than in the control group under all conditions with moderated to large effect size (floor: *p* = 0.014, *r* = 0.413; BOSU: *p* = 0.008, *r* = 0.441; balance ball: *p* < 0.001, *r* = 0.586) (Figure 6). The ratio of lumbar multifidus muscle activity to erector spinae muscle activity was significantly lower in the NSCLBP group than in the control group in the balance ball condition with a large effect size (*p* < 0.001, *r* = 0.597), but no significant difference was found in the floor or BOSU conditions with small to moderated effect size (*p* = 0.067–0.118) (Figure 7).

## 4. Discussion

This study examined the differences in the effects of unstable conditions on trunk muscle activity during exercise in the NSCLBP and control groups. The NSCLBP group showed a significantly greater increase in erector spinae muscle activity from the floor condition to the balance ball condition than the control group, although neither group showed any significant change in trunk muscle activities in the BOSU condition, compared with those in the floor condition. The changes in internal oblique/transversus abdominis muscle activity between the floor and balance ball conditions were not significant for the NSCLBP group but were significant for the control group. To the best of our knowledge, this is the first study to show that changes in trunk muscle activity induced by the balance ball condition are different between NSCLBP and healthy individuals. These findings suggest that exercise therapy using the balance ball should be a caution in the management of NSCLBP.

In the present study, we showed that the erector spinae muscle activity during the supine bridge exercise using the balance ball, normalized by the floor supine bridge exercise, was significantly greater in the NSCLBP group, and the internal oblique/transversus abdominis muscle activity was significantly lower than in the control group. It has been reported that individuals with LBP show inactivity in the local muscles [7,12,13,14] and increased activity in the global muscle groups [12,13,14,34,35,36,37]. The amplitude of transversus abdominis activity was significantly decreased, and the onset timing of transversus abdominis muscle and internal oblique/transversus abdominis muscle was significantly delayed during rapid upper and lower limb movement in individuals with NSCLBP compared to the healthy individuals [8,9,10,11]. The transversus abdominis muscle attaches to the thoracolumbar fascia and is thought to contribute to spinal intersegmental rigidity by exerting lateral tension on the thoracolumbar fascia [34]. In addition, the activity of the transversus abdominis muscle is thought to increase intra-abdominal pressure, thus contributing to the rigidity of the interspinal segments [35,36]. Also, it has been suggested that the internal oblique muscles may play a role like that of the transversus abdominis and may similarly contribute to stiffness between spinal segments [37]. These local muscle dysfunctions may lead to spinal intersegmental instability. Besides, in the global muscle, it has been reported that the lumbar and thoracic erector spinae exhibit a higher amplitude of activity during active trunk movements in individuals with NSCLBP than the healthy controls [8]. Increased global muscular activity increases spine stress [38], impairing spine mobility and contributing to shock absorption/damping [39]. The results of this study support the findings of these previous studies [8,9,10,11,12,13,14,34,35,36,37]. These inappropriate recruitment patterns may contribute to NSCLBP [9]. To resolve such dysfunction and pain, exercises that emphasize local muscle activity during rehabilitation for patients with NSCLBP have been suggested [17]. The results of this study indicated that exercising using the balance ball emphasizes inappropriate muscle recruitment patterns in patients with NSCLBP. It is necessary to be aware of these changes in trunk muscle activities when patients with NSCLBP perform exercises using the balance ball.

The age difference may have a potential impact on group differences in trunk muscle activity. However, no significant group difference in age was detected in this study. Therefore, the effect of age on the group differences in muscle activity would be minimal. In this study, the activities of most muscles normalized by MVIC in both groups were significantly greater in the balance ball condition than in the floor condition. These increases in muscle activities may be due to the plasticity of the balance ball. The balance ball condition needs more muscle activity than the floor condition because the balance ball is softer and deforms with external forces, making it harder to generate a reaction force. Wahl et al. reported increased trunk muscle activity during standing and squatting with an unstable device, especially those with a smaller base of the support surface and greater malleability [40].

We also investigated trunk muscle activities during supine bridge exercises under another unstable condition using the BOSU balance trainer. In contrast to the balance ball condition, when the activity normalized by MVIC was compared between the BOSU and floor conditions, there were no significant differences in all muscle activity in both groups. In addition, there were no significant differences between the groups in the trunk muscle activities in the BOSU condition normalized by the floor supine bridge exercise. A previous study reported no significant difference in trunk muscle activity during supine bridge exercises between floor and BOSU conditions [26]. Therefore, these findings suggest that the application of the BOSU balance trainer to supine bridge exercises might not create a condition unstable enough to affect trunk muscle activities.

In the present study, the ratio of lumbar multifidus activity to erector spinae was significantly lower in the NSCLBP group than in the control group in the balance ball condition. This result supports our finding that spinal erector spinae muscle activity in the balance ball condition, normalized by muscle activity on the floor, is greater in NSCLBP patients than in controls. After all, exercise using the balance ball in patients with NSCLBP can emphasize inappropriate muscle recruitment patterns, and clinicians should be aware of changes in trunk muscle activity. Our study also showed that the ratio of internal oblique/transversus abdominis muscle activity to rectus abdominis muscle activity was significantly lower in the NSCLBP group than in the control group under all conditions. This result indicates that abdominal muscle imbalance occurs in individuals with NSCLBP, regardless of whether the condition is unstable or not. A previous study reported that patients with LBP cannot preferentially work the transversus abdominis muscle over the rectus abdominis muscle [41]. A previous study reported an improvement in the ratio of internal abdominal oblique muscle activity to rectus abdominis muscle activity in patients with LBP after 10 weeks of exercise therapy, focusing on local muscle activity [18]. The results of local to global muscle activity ratio suggest that exercises to increase local muscle activity must first be performed. On the other hand, there was no significant difference in the ratio of internal oblique/transversus abdominis muscle activity to external oblique muscle activity between the groups in all conditions. A previous study has shown that the demand for external oblique is greater in exercises that occur when a rotational moment of the spine is expected, such as a single bridge with one leg elevated [19]. The present study examined muscle activity during symmetrical exercises, which may not have provided sufficient muscle activity for intergroup differences to exist.

In terms of clinical significance, this study showed the different changes in trunk muscle activity during exercises caused by the balance ball condition in patients with NSCLBP. Exercise therapy is recommended as a conservative treatment for NSCLBP [20,21,22], and many studies have shown that exercise using the balance ball requires higher muscle activity in healthy individuals [25,26,27]. However, for the treatment of patients with NSCLBP, exercises that focus on the local muscle group, such as motor control exercises, are considered effective [42,43,44]. Tsao et al. reported that motor control exercises changed the onset timing of activity in the transversus abdominis muscle and that the intervention effect persisted 6 months later [45]. Additionally, the motor control exercise intervention has also been shown to decrease the activity of the global trunk muscle group in trunk active movements, lifting, and trunk external perturbation tasks [46,47,48]. Recent randomized control trials have reported that motor control exercise reduces pain intensity and functional impairment in NSCLBP, in addition to changes in the morphology of the trunk local muscle groups [49]. Therefore, the strategy of exercise therapy for NSCLBP would be effective in activating the local muscles and inhibiting the global muscles. The present study showed that participants with NSCLBP mainly increased erector spinae muscle activity during the bridge exercise using the balance ball. However, there were no significant changes in the activity of the internal oblique/transverse abdominal muscles between the floor and the balance ball condition. Richardson et al. suggested that as the intervention progresses, there should be a transition to increasingly challenging exercises that involve the activation of both local and global muscles [17]. For patients with NSCLBP, it may be advisable to first perform exercises focusing on the local trunk muscle groups, followed by exercises on the floor after sufficient activity is confirmed, and finally, exercises that require high muscle activity, such as using a balance ball. This study suggests that caution is needed in the management of NSCLBP and in performing exercises using the balance ball.

This study had some limitations to be addressed. First, the participants in each group were limited to young individuals, even though individuals with NSCLBP are not limited to young individuals. This limits the generalizability of the present findings to a broader age range, as the impact of NSCLBP and exercise therapy might vary across different age groups. Secondly, this study examined differences in the effects of unstable surfaces on trunk muscle activity during exercise in the NSCLBP and control groups but only examined the immediate effects of bridge exercises. The bridge exercise was employed because it is frequently used in rehabilitation [25,26,27]. However, other exercises and long-term continuation of exercises may have different effects. Thirdly, EMG recordings were performed using a surface EMG system. Although the electrode positions in this study were in accordance with those of previous studies [26,30,31], the use of surface EMG may detect activity other than the target muscles, especially for specific deep muscles. Intramuscular EMG might provide more precise data. However, it is often more invasive, which can affect muscle activity. Fourthly, MVIC was used to normalize the muscle activity to compare muscle activity during supine bridge exercises on the floor and in unstable conditions in each group. Using MVIC for normalization might pose challenges in individuals with NSCLBP, as they may have difficulty performing MVIC reliably. This could affect the accuracy of the normalization process. However, there is still no established consensus on the most suitable normalization procedure for individuals with NSCLBP [50]. Fifthly, the mean Oswestry Disability Index was 14.2, and the numerical rating scale was 3.1. It has been reported that the mean ODI score of Japanese people in their 20s who suffer from low back pain and have difficulty performing daily work is 15.86 [51]. The mean ODI values in this study were lower than those in the previous study, suggesting that participants in our NSCLBP group had a milder intensity of pain. Therefore, the results of this study may be generalizable to participants with mild NSCLBP. Sixthly, although a rigorous control of confounding variables is important in conducting a case-control study such as this study, it is possible that some potential confounding variables like profession and lifestyle may have influenced the results in this study.

## 5. Conclusions

The present study showed that muscle activity of the global back muscle during supine bridge exercises using the balance ball was significantly greater in participants with NSCLBP than in those without NSCLBP, while that of abdominal local muscle was significantly smaller in participants with NSCLBP than in those without NSCLBP. Considering that local muscle dysfunctions lead to spinal intersegmental instability and increased global muscular activity impairs contribution to shock absorption/damping, exercises using the balance ball should be used with caution in the rehabilitation of NSCLBP.

## Figures and Tables

**Table 1:** Participant characteristics in non-specific chronic low back pain and control groups.

	NSCLBP Group (n = 17)	Control Group (n = 18)	*p*-Value	Effect Size
Age, yrs	21.8 (2.6, 19–29)	21.6 (1.0, 20–23)	0.245 ^a^	0.208 ^a^
Height, cm	166.5 (5.5, 156.4–176.1)	164.8 (8.3, 156.4–179.5)	0.522	0.241
Weight, kg	60.6 (7.8, 47.0–71.1)	56.3 (8.2, 43.0–67.0)	0.106	0.537
BMI, kg/m^2^	21.8 (1.9, 19.2–25.4)	20.6 (1.9, 17.8–24.7)	0.061	0.632
NRS, /10	3.1 (1.7, 2–7)	-	-	-
ODI, %	14.2 (9.2, 2.5–26.0)	-	-	-
Duration of pain, yrs	5.3 (0.25–10)	-	-	-

The values are presented as means (standard deviation, range). ^a^ The data were not normally distributed, nonparametric tests were used, and *r* was used for the effect size. Abbreviations: NSCLBP—non-specific chronic low back pain; BMI—body mass index; NRS—numeric rating scale; ODI—Oswestry disability index.

**Table 2:** The effect size for comparison of trunk muscle activity normalized by maximum voluntary isometric contraction between balance ball and floor condition in each group.

	Effect Size	
	NSCLBP Group (n = 17)	Control Group (n = 18)
Internal oblique/transversus abdominis	0.465 ^a^	0.549 ^a^
External oblique	0.718 ^a^	0.652 ^a^
Rectus abdominis	0.591 ^a^	0.652 ^a^
Erector spinae	0.729 ^a^	0.760
Multifidus	0.752 ^a^	0.739

^a^ The data were not normally distributed and *r* was used for the effect size.

## Data Availability

The data that support the findings of this study are available from the corresponding author upon reasonable request.
